# Beyond Composition: Processing-Driven Transformations and Functional Trade-Offs of Matcha Tea in Foods

**DOI:** 10.3390/foods15162782

**Published:** 2026-08-07

**Authors:** Alev Onder, Mohamad Warda, Ozge Yılmaz, Jaehoon Bae, A. M. Abd El-Aty

**Affiliations:** 1 Department of Pharmacognosy, Faculty of Pharmacy, Ankara University, 06100 Ankara, Türkiye; ozgeadaletyilmaz@gmail.com; 2 Department of Physiology, Faculty of Veterinary Medicine, Atatürk University, 25240 Erzurum, Türkiye; mohamad.warda@atauni.edu.tr; 3 Department of Biochemistry and Molecular Biology, Faculty of Veterinary Medicine, Cairo University, Giza 12211, Egypt; 4 Functional Food Research Institute, Industry-University Cooperation Foundation, Daegu Haany University, Gyeongsan 38610, Republic of Korea; 5 Department of Pharmacology, Faculty of Veterinary Medicine, Cairo University, Giza 12211, Egypt; abdelaty44@hotmail.com; 6 Department of Medical Pharmacology, Faculty of Medicine, Ataturk University, 25240 Erzurum, Türkiye

**Keywords:** matcha tea, food matrices, processing-induced transformations, catechins, bioaccessibility

## Abstract

Matcha tea, a powdered green tea produced from shade-grown *Camellia sinensis* leaves, has gained increasing attention as both a traditional beverage and a functional food ingredient. While previous reviews have focused on its phytochemical profile and biological activities, less attention has been given to the effects of food processing and matrix interactions on matcha bioactives. This review critically synthesizes current evidence on the processing-induced transformation, stability, and digestive bioaccessibility of key matcha compounds in real food systems. The production methods, phytochemical compositions, and reported health effects are summarized, with a particular emphasis on matrix-dependent changes during processing, including catechin degradation and epimerization, caffeine retention, L-theanine stability, and chlorophyll loss. The implications of these alterations for sensory quality, safety, and gastrointestinal release are discussed. Emerging findings on gut microbiota modulation and omics-based approaches are also highlighted. Collectively, the available evidence indicates that the functional value of matcha in foods is strongly influenced by processing–matrix interactions rather than native composition alone, leading to trade-offs among health functionality, consumer acceptance, and safety. By integrating food chemistry, processing, and nutritional perspectives, this review proposes a framework to guide the rational formulation of matcha-enriched foods and align health claims with realistic processing outcomes.

## 1. Introduction

Functional foods are derived from plants or animals and contain essential phytochemicals, which offer both physiological effects and nutritional benefits. Tea, one of the oldest and most widely consumed brewed beverages, is made from various parts of the tea plant, including its shoots, stems, and leaves. The scientific name of the tea plant is *Camellia sinensis* (L.) Kuntze, and it belongs to the Theaceae family. It is considered one of the most important functional foods [1].

The history of the tea plant dates back approximately 5000 years, linked to a “Zen Buddhist” legend that suggests that tea was used as a stimulant and for spiritual cleansing [2]. Over the past seven centuries, tea has become popular worldwide [3]. The use of tea as a beverage started during the Chinese Emperor Shen Nung’s reign and gradually spread beyond the aristocracy, first throughout China and then to Japan [3,4], as well as to Indonesia and India. The British played a major role in the tea trade during the 17th century, helping it gain widespread popularity, and tea was even used as a form of currency because of its international value [3].

In many regions, drinking tea is seen as an enjoyable activity that reflects cultural traditions because of its status as a healthy, safe, and affordable beverage [4]. The chemical composition of *Camellia sinensis* contains more than 4000 phytochemicals, with more than 700 known for their beneficial effects on human health [5]. Regular tea consumption has been linked to a wide range of preventive effects against diseases, including coronary heart disease, obesity, diabetes, inflammatory conditions, and infectious diseases [1,6].

There are five types of tea made from *Camellia sinensis*, white, green, oolong, black, and Pu-erh, depending on the fermentation level and production stage [7]. Green and oolong teas are commonly consumed in Asian countries, especially China and Japan. In contrast, black tea consumption varies in other regions, often depending on brewing methods, and is usually sweetened with sugar, milk, honey, or lemon [3,8]. Green tea, which has a yellowish-green color, a light aroma, and a bitter-astringent taste, is prepared without fermentation [9].

Matcha tea is a special type of green tea that is traditionally present in Japanese tea ceremonies and is obtained by grinding the leaves of one of the green tea varieties called Tencha [10]. The use of matcha tea in tea ceremonies dates back to the 13th century, when it was employed by Japanese Zen priests to increase their concentration and relaxation during extended meditation sessions [11]. The matcha tea used in traditional ceremonies is of the highest quality; more economical and culinary matcha tea is used as a food ingredient [12]. In addition to being used as a food ingredient, matcha tea is also included in many cosmetic products used for hygiene and personal care [13]. The production stages of green tea include sequential sun-drying, tumble-rolling, and steaming. Although matcha tea is a type of green tea, the production stages differ in that the tea leaves are exposed to shade and ground approximately 1 month before harvest. These differences in production stages result in matcha tea being brighter green than traditional green tea, which has greater nutritional value [14].

It is important to include matcha tea in this review, as fewer studies have focused on it than on green tea. Additionally, its popularity and importance in the food industry are increasing. Therefore, this review aims to summarize the research on the production process, phytochemicals, biological activity, nutritional value, and role of matcha tea in the food industry on the basis of the available literature.

Beyond its traditional production, contemporary matcha manufacturing integrates controlled shading before harvest, steam fixation to prevent enzymatic oxidation, tencha production, and fine milling technologies to obtain the characteristic powder. These processing steps are recognized as major determinants of the chlorophyll, catechin, caffeine, and L-theanine contents of matcha and therefore influence its nutritional quality and functional performance in food applications. Consequently, processing conditions have become important when developing matcha-containing foods and evaluating their physicochemical and biological properties [15,16,17].

For this review, relevant literature was retrieved from PubMed, Scopus, and Web of Science using combinations of keywords related to matcha processing, *Camellia sinensis*, catechins, chlorophyll, caffeine, L-theanine, bioactive compounds, functional foods, and health effects. Studies published between 1997 and 2026 were considered to provide an updated overview of current knowledge regarding matcha production, composition, and applications. This narrative review primarily considered peer-reviewed studies relevant to matcha tea in food systems, with emphasis placed on investigations addressing processing-induced transformations, food matrix interactions, digestive bioaccessibility, and functional outcomes of matcha bioactives. Particular emphasis was placed on recent studies and evidence derived from food-relevant experimental models, including *in vitro*, *in vivo*, and clinical investigations. The available evidence was critically appraised according to study design, methodological rigor, consistency of findings across reports, and relevance to real food applications.

Several reviews have addressed the chemical composition, processing characteristics, and health-promoting properties of matcha tea; however, the behavior of its bioactive constituents after incorporation into complex food matrices remains insufficiently explored [16,17,18]. The functional performance of matcha in foods cannot be predicted solely from its native composition, as processing conditions and interactions with other food components may influence the stability, transformation, release, and bioaccessibility of key compounds. Therefore, a processing–transformation–function framework is proposed in this review to examine how thermal treatments, storage conditions, and matrix interactions affect the fate of matcha-derived bioactives and their subsequent implications for nutritional value, sensory attributes, and food safety.

In contrast to previous reviews that have focused mainly on the composition or biological effects of the individual constituents of matcha, this review provides a critical evaluation of the bioactive stability, processing-induced transformations, and digestive bioaccessibility of matcha within real food systems. Particular attention is given to the balance between bioactive compound preservation, technological functionality, sensory acceptance, and safety requirements during food formulation. Accordingly, the scope of this review is limited to food-based applications of matcha and excludes pharmaceutical preparations and studies based on isolated compound supplementation.

To delineate the theoretical scope of this review relative to the literature, we conducted a systematic comparative gap analysis across recent major matcha syntheses [15,16,17,18]. Prior reviews have predominantly examined matcha through a classical pharmacological or raw-compositional lens, focusing primarily on isolated bioactive identification or broad health outcomes in beverage/infusion models. However, these frameworks systematically overlook the behavior of matcha within real, complex food systems. To bridge this gap, this review establishes a processing–transformation–function paradigm evaluated across seven critical food-matrix parameters:

1. Processing Stability of Bioactives (PSB)
2. Food Matrix Interactions (FMIs)
3. Catechin Degradation and Epimerization (CDE)
4. Caffeine–theanine functional balance (CTFB)
5. Sensory and Acceptance Trade-offs (SCTo)
6. Digestive Bioaccessibility in Food Matrices (DBF)
7. Applied Safety Considerations (SC)

As synthesized in Table 1, existing reviews address these parameters either restrictively (e.g., limited to aqueous infusions) or not at all. Table 1 thus serves as the foundational analytical matrix for this manuscript, defining the multiparameter space required to evaluate matcha when formulated into solid and semisolid food applications. The subsequent sections of this review are explicitly structured around these seven key dimensions to systematically map processing-induced functional trade-offs.

## 2. Production of Matcha Tea

Matcha tea is derived from *Camellia sinensis*, which is grown under shaded conditions for an average of 20–30 days before harvesting. During this process, the stems and veins are removed from the leaves, which are then ground into a fine, shiny green powder. This production method sets matcha apart from other green teas [14]. Owing to its cultivation under shade, matcha contains relatively high levels of chlorophyll, amino acids, and caffeine, contributing to its distinct “umami” flavor, which is particularly enhanced by its elevated amino acid content [10,19,20]. The “umami” flavor profile of tea is influenced by the balance between catechins and theanine. When matcha tea is compared with regular green tea, 0.5 g of green tea results in a bitterness similar to that of approximately 1.5 g of matcha tea [14].

Shade cultivation is among the most critical determinants of matcha raw material quality. Tea plants are typically covered with shade nets that reduce sunlight by approximately 70–95% for the final 20–30 days before harvest [21,22]. The reduced light intensity suppresses catechin biosynthesis [21], while promoting the accumulation of chlorophyll, L-theanine, and other free amino acids, resulting in the characteristic bright green color, enhanced umami taste, and lower astringency of high-quality matcha. The extent and duration of shading therefore directly influence the chemical composition and sensory properties of matcha, although the final quality grade also depends on cultivar selection, harvest timing, and subsequent processing [22].

Matcha production involves several sequential processing steps after harvest. Fresh leaves are rapidly steam-treated to inactivate oxidative enzymes, such as polyphenol oxidase, and preserve their green color and phytochemical composition before being dried to produce tencha [11,23]. The dried leaves are subsequently destemmed and deveined, after which they are milled into an ultrafine powder [11]. Traditionally, premium matcha is produced using slow granite stone mills, which minimize heat generation and preserve chlorophyll, volatile compounds, and particle uniformity [11,24]. In contrast, modern industrial production increasingly employs ceramic, jet, or ball milling systems that improve manufacturing efficiency but may generate greater frictional heat, potentially influencing particle size, color stability, aroma, and the retention of bioactive compounds [24]. Consequently, milling technology represents an important determinant of final matcha quality. Overall, cultivar selection, shading conditions, harvest timing, steam fixation, and milling technology collectively affect the physicochemical composition, sensory characteristics, and functional quality of matcha.

## 3. Consumption of Matcha Tea

When matcha tea is being prepared, it is important to avoid the use of boiling water, as boiling water can breakdown its phytochemicals and reduce its nutritional value. Traditional matcha preparation requires a bamboo scoop (chashaku), a tea bowl (chawan), and a bamboo whisk (chasen). In traditional Japanese tea ceremonies, matcha is made by adding hot water at approximately 70 °C to tea via a bamboo spoon called a “Shashaku”. This mixture is then placed in a preheated tea bowl, known as a “Chawan,” and whisked with a bamboo whisker until it has a smooth consistency [20]. Matcha can be prepared with different strengths, such as regular matcha, usucha (dilute), or koicha (dense), depending on the ratio of tea to water [19,20]. Unlike regular green tea, matcha is not filtered, which means that the entire tea leaf is consumed. This process allows for greater intake of beneficial bioactive compounds [24].

In addition to its traditional preparation as a whisked beverage, matcha is now consumed in a wide variety of forms owing to its versatility as a functional food ingredient [25,26]. It is frequently incorporated into dairy products, including milk-based beverages, yogurts, and ice cream, as well as bakery products such as cakes, cookies, bread, and pastries [25]. Matcha is also widely used in confectionery, chocolates, smoothies, ready-to-drink beverages, nutritional supplements, and powdered drink mixes [25,26]. Furthermore, it has gained increasing popularity as an ingredient in functional foods because it provides color, flavor, and bioactive compounds while enabling the development of products with enhanced antioxidant potential [26,27]. The choice of consumption format may influence the stability, bioaccessibility, and sensory perception of matcha bioactive compounds because different food matrices and processing conditions can modify their release and functionality [26,28].

## 4. Phytochemical Content

**Figure 1:**
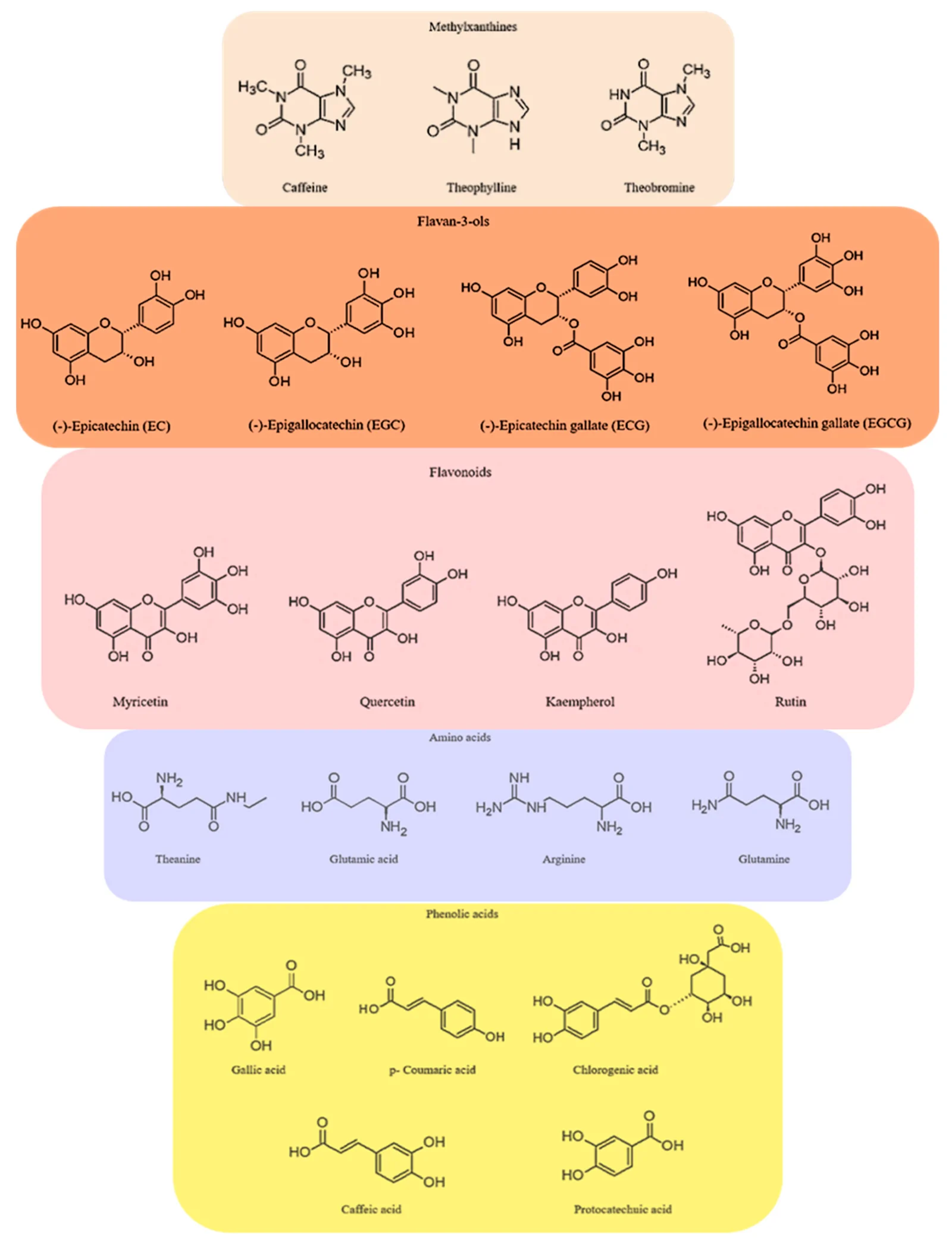
The main chemical contents of matcha tea.

Matcha tea is a unique type of green tea whose phytochemical content is similar to that of regular green tea [13,29,30,31]. While the phytochemical composition of matcha tea closely resembles that of green tea, the proportions of specific components differ. Matcha tea is rich in catechins, flavonoids, and phenolic acids, which are collectively known as tea polyphenols. It also contains notable amounts of theanine, chlorophyll, and caffeine [19]. A study revealed that matcha tea includes various compounds (Figure 1), such as (-)-gallocatechin (GC), (-)-epigallocatechin (EGC), (-)-epigallocatechin gallate (EGCG), (-)-epicatechin (EC), catechin, (-)-gallocatechin gallate (GCG), (-)-epicatechin gallate (ECG), caffeine, theobromine, theophylline, *p*-coumaric acid, protocatechic acid, gallic acid, and ferulic acid [32].

In a study by Sayuti et al., high-performance liquid chromatography (HPLC) analysis of matcha tea samples revealed that the extract contained EGCG, ECG, catechin, rutin, tannic acid, myricetin, chlorogenic acid, gallic acid, EC, cinnamic acid, rosmarinic acid, luteolin, and kaempferol at the highest to lowest concentrations [33]. Comparisons of catechin amounts between aqueous green tea and methanolic extracts of matcha tea via micellar electrokinetic chromatography indicated that matcha tea has relatively high levels of catechins, particularly (-)-epigallocatechin-3-gallate (EGCG). Additionally, a previous study revealed that the aqueous extract of matcha tea presented the highest caffeine content [34].

Koláčková et al. (2020) [19] reported that matcha tea samples contain several beneficial compounds, including gallic acid, chlorogenic acid, caffeic acid, ellagic acid, protocatechic acid, p-hydroxybenzoic acid, ferulic acid, sinapic acid, rutin, quercetin, kaempferol, caffeine, vitamin C, chlorophyll, protein, lipids, and fiber. The organic and fatty acids in these matcha tea samples were analyzed via gas chromatography, after which oxalic, malic, citric, and succinic acids were identified. Low-quality matcha tea samples presented decreased levels of citric acid and increased levels of oxidized unsaturated fatty acids [35]. Moreover, Kika et al. (2024) [15] reported that compared with saturated fatty acids, matcha tea has a greater concentration of unsaturated fatty acids, with α-linolenic acid being the predominant unsaturated fatty acid and palmitic acid being the major saturated fatty acid. This study also revealed that the main components of matcha tea included caffeine, EGC, and gallic acid, along with various flavonoids and phenolic acids, although chlorogenic acid and caffeic acid were not present [15]. This apparent discrepancy between these findings likely reflects differences in matcha cultivar, geographical origin, processing conditions, and analytical methods employed among studies. The concentrations of minor phenolic compounds, such as chlorogenic and caffeic acids, may vary substantially among matcha samples and can also be influenced by extraction procedures and analytical detection limits. Therefore, the absence of these compounds in certain studies should not necessarily be interpreted as evidence that they are universally absent from matcha tea.

An investigation into the effect of drying under shade on the phytochemical composition of matcha tea obtained from Assam tea, *C. sinensis* var. *assamica* (JWMasters) Kitamura, revealed that the number of phenolic compounds, catechins, and flavonoids changed inversely with the shading rate. Nevertheless, the amount of protein did not change significantly. Consumers generally prefer matcha tea, which is produced with greater shading. This preference is likely due to the reduction in catechin levels, which lessens the bitter taste that catechins can impart. However, while increased shading may increase consumer appeal, it has been reported to negatively affect the bioactivity of matcha tea. Specifically, this shading can lead to a decrease in phenolic compounds, particularly those responsible for antioxidant activity, which are known to provide various health benefits [36]. According to one study, the total protein content of matcha tea was determined to be 17.3 g/100 g [15]. Although matcha is not typically consumed in quantities sufficient to constitute a major dietary protein source, its protein content may contribute modestly to the nutritional profile of matcha-containing foods and beverages. Furthermore, free amino acids play a significant role in determining the quality of matcha tea. A comprehensive analytical study investigated the amino acids and phenolic compounds in matcha tea samples of varying quality grades. High-quality matcha tea samples presented relatively high amino acid levels, along with relatively low catechin levels. Conversely, lower-quality matcha samples presented higher levels of catechin and lower levels of free amino acids [37]. The most abundant free amino acid was theanine, followed by glutamic acid, γ-aminobutyric acid (GABA), methionine, and threonine, while the bound amino acids were glutamic acid, aspartic acid, leucine, lysine, and arginine [19]. Finally, the structures of the phytochemicals present in matcha tea are shown in Figure 1.

While the phytochemical profile of matcha tea provides a foundation for its functional potential, the mere presence of catechins, theanine, chlorophyll, and caffeine does not fully determine their physiological impact. Once ingested, these compounds undergo digestive transformations and interact extensively with the gut microbiota, modulating their bioaccessibility and downstream biological effects. Therefore, understanding how matcha bioactives are metabolized and transformed within the gastrointestinal tract is essential for linking composition to health outcomes. The following section explores the microbiota-mediated metabolism of catechin epimers, chlorophyll derivatives, and caffeine, as well as the matrix-dependent factors that shape their bioaccessibility and signaling activity.

## 5. The Gut Microbiota and Bioaccessibility

### 5.1. Catechin Epimers and Microbial Metabolism

Catechins undergo extensive microbial biotransformation in the colon, yielding relatively small amounts of phenolic acids and related metabolites that contribute to systemic antioxidant and anti-inflammatory effects by increasing their bioavailability and biological activity beyond those of the parent compounds. The gut microbiota-mediated metabolism of green tea catechins results in the production of phenyl-γ-valerolactones, phenolic acids, and other low-molecular-weight metabolites that may be absorbed or contribute locally to gut health [38]. *In vitro* studies have indicated that human gut microbes degrade catechins through ester hydrolysis, ring opening, and dehydroxylation, leading to the formation of phenolic acids that have been demonstrated to increase antioxidant bioactivity [39]. Interactions between dietary flavonoids and the gut microbiome suggest that the microbial metabolism of catechins in the colon can influence microbial community structure as well as metabolic outputs [40]. Epimerized catechins such as gallocatechin gallate may also be metabolized by distinct gut bacteria, potentially altering fermentation patterns and the production of short-chain fatty acids (SCFAs). SCFAs—including acetate, propionate, and butyrate—are key microbial metabolites with demonstrated roles in host energy metabolism, immune regulation, and maintenance of gut barrier integrity [41].

Nevertheless, the available evidence remains subject to several limitations. Much of the current understanding of catechin biotransformation is derived from *in vitro* fermentation models and preclinical studies, which may not fully capture the complexity of the human gastrointestinal environment [42,43]. Moreover, considerable interindividual variability exists in the gut microbiota composition and metabolic capacity, which may influence the extent of catechin degradation, the profile and abundance of microbial metabolites produced, and their subsequent physiological effects [42,44]. Factors such as age, dietary habits, geographical location, health status, and antibiotic exposure can substantially alter microbial community structure and consequently affect the bioaccessibility and metabolism of matcha-derived catechins [45,46]. Therefore, the health outcomes associated with catechin consumption may vary among individuals, highlighting the need for additional well-designed human studies to elucidate microbiota-dependent responses and their implications for personalized nutrition [42,43].

### 5.2. Chlorophyll Breakdown Products

Chlorophyll degradation during food processing yields derivatives such as pheophytins and pyropheophytins, which differ structurally from native chlorophyll and have distinct digestive and microbial fates. During *in vitro* gastrointestinal digestion and colonic fermentation, chlorophyll is extensively converted to pheophytin a, pheophytin b, and other metabolites, and these derivatives influence gut microbial fermentation dynamics, increasing total short-chain fatty acids, including acetate, propionate, and butyrate [47]. The digestive stability and absorption pathways of chlorophyll derivatives vary with structure, with dephytylated forms showing altered micellar behavior and intestinal uptake [48]. Emerging evidence also indicates that chlorophyll and related derivatives can modulate the gut microbiota composition and host metabolic responses, including effects on inflammation and glucose or lipid metabolism, which are mechanistically linked to microbiota–bile acid interactions [49].

### 5.3. Interactions Between Caffeine and the Microbiota

Caffeine, which is abundant in matcha, is metabolized by both host enzymes and gut microbes, and emerging evidence indicates that caffeine consumption can shape the gut microbial composition and diversity. In high-fat diet-fed mice, caffeine supplementation increased the relative abundance of genera such as *Bifidobacterium* and *Desulfovibrio* while decreasing the abundance of *Bacteroides* and *Lactobacillus* and altering bile acid and energy metabolism [50]. Human data have shown that higher caffeine intake is correlated with greater alpha diversity of the colonic microbiota and increases in taxa such as *Faecalibacterium* and *Alistipes* [51]. Moderate caffeine-containing beverage consumption has also been linked to increases in beneficial bacteria, including *Bifidobacterium* spp., and increased overall diversity [52]. These interactions suggest that caffeine may synergize with other tea bioactive compounds, such as catechins, to influence the gut ecosystem beyond its effects on the central nervous system, potentially contributing to metabolic and immune regulation through microbiota-mediated pathways.

### 5.4. Bioaccessibility and Host Signaling

The digestive bioaccessibility of matcha bioactives is strongly influenced by food matrix interactions. Catechin–protein complexes in dairy, starch encapsulated in bakery products, and lipid emulsions in confectionery all alter the release kinetics of bioactive compounds, shifting their sites of microbial interaction during gastrointestinal digestion [28]. Polyphenols that survive upper-gut digestion reach the colon, where they are extensively transformed by the gut microbiota into lower-molecular-weight metabolites that can exert biological effects beyond their antioxidant capacity [53]. These microbiota-derived metabolites have been linked to the modulation of key host signaling pathways, including the AMPK, NF-κB, and PPARγ pathways, thereby providing a plausible mechanistic link between matrix-dependent bioaccessibility and systemic metabolic and inflammatory regulation [54]. Thus, matcha may function not only as a direct antioxidant but also as a prebiotic-like modulator of host–microbe crosstalk. However, the currently available evidence supporting these mechanisms is derived largely from *in vitro* experiments, animal models, and studies of individual polyphenol metabolites rather than whole matcha preparations. The magnitude and direction of host signaling responses may also depend on interindividual differences in gut microbiota composition, dietary context, and the bioaccessibility of matcha-derived compounds. Consequently, although modulation of AMPK, NF-κB, and PPARγ signaling pathways represents a plausible mechanistic link between microbial metabolism and host physiology, additional well-controlled human studies are needed to establish causal relationships and determine their clinical relevance.

**Figure 2:**
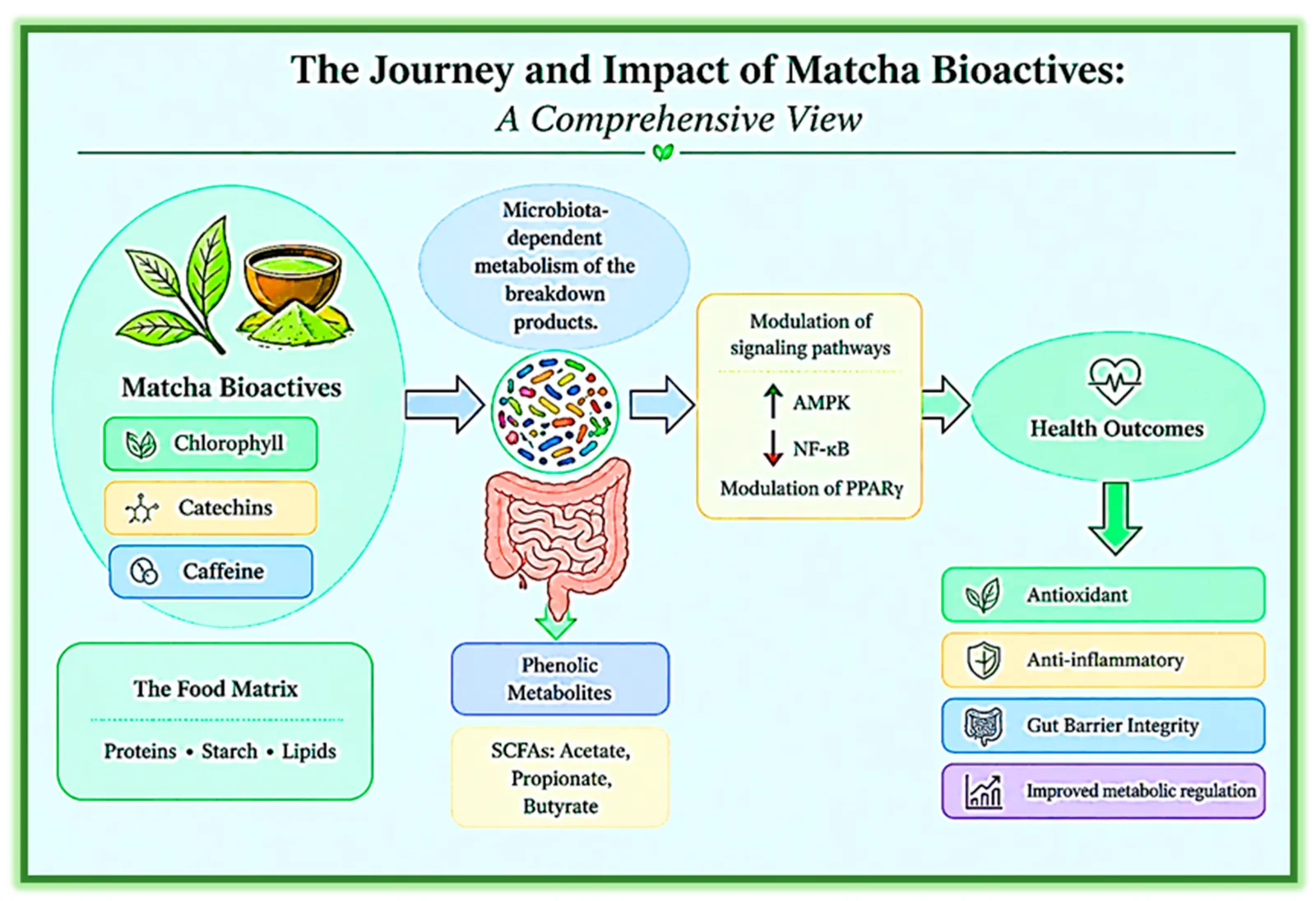
Schematic representation of the journey and impact of matcha bioactives on the gut microbiota and host signaling. Matcha-derived compounds (catechins, caffeine, and chlorophyll) undergo matrix-dependent digestive transformations and microbial metabolism, yielding short-chain fatty acids (acetate, propionate, and butyrate) and phenolic derivatives. These metabolites modulate critical host signaling pathways, including AMPK, NF-κB, and PPARγ, thereby linking dietary intake to systemic antioxidant, anti-inflammatory, and gut barrier–protective outcomes. The illustration highlights the sequential relationships among food matrix interactions, microbial biotransformation, and health functionality, clarifying the bioaccessibility–microbiota–host axis.

These interconnected processes are summarized schematically in Figure 2, which illustrates the proposed bioaccessibility–microbiota–host signaling axis underlying the potential health effects of matcha bioactives.

## 6. Biological Activities

Owing to the higher concentration of bioactive compounds found in matcha tea than in green tea, the use of matcha tea as an unfiltered beverage is expected to provide greater health benefits [55]. Matcha tea has been shown to possess antioxidant, antimicrobial, anti-inflammatory, anticancer, and neuroprotective properties. Additionally, it has positive effects on metabolic syndrome, weight management, and gut health because of its potent components [16,18].

### 6.1. Antioxidant and Anti-Inflammatory Activities

Matcha tea samples were found to have approximately twice the total phenolic content as green tea and white tea samples at all infusion temperatures and greater iron-reducing antioxidant activity. While preparing matcha tea with a maximum water temperature of 80 °C is recommended, some matcha tea samples prepared with 75 °C and 90 °C water also present strong antioxidant effects. In addition, the most suitable extraction conditions for phenolic compounds in matcha tea were 80 °C water for 20 min and a liquid-to-solid ratio of 100 mL/g [33]. A study by Koláčková et al. revealed that the vitamin C content of matcha tea samples was twice that of other tea types. However, vitamin C did not contribute to the antioxidant effects of chlorophyll and caffeine in aqueous extracts of matcha tea samples enriched with 80% methanol, which might be related to the total phenolic and flavonoid contents [19]. The effect of *in vitro* digestion on matcha tea emphasized that the antioxidant activity of matcha tea after digestion was 6%, indicating that 94% of its antioxidant effect could be retained by digestion. The major phenolic compounds detected in the nondigestible part of matcha tea, such as rutin, EC, chlorogenic acid, and ellagic acid, are not digested and pass into the large intestine in humans [26]. In a comparison study, the green tea samples contained more catechins and presented greater antioxidant activity than the matcha tea samples did.

Moreover, compared with the green tea samples, the matcha tea samples contained more caffeine; however, the culinary matcha tea samples presented a higher phenolic content than did the ceremonial matcha tea samples did. For this reason, ceremonial matcha tea has relatively low antioxidant activity [12]. The concomitant administration of *Spirulina platensis* and matcha tea (3 mg/kg, for six weeks) has been demonstrated to have curative effects through its antioxidant and anti-inflammatory properties. This effect was observed in response to a parasitic infection, schistosomiasis, caused by *Schistosoma mansoni*, in liver and spleen tissues [56]. In another study, the antioxidant and anti-inflammatory effects of matcha tea silver particles (M-AgNPs) were investigated in rats exposed to γ-ray radiation. M-AgNPs increased antioxidant defense at the cellular level by increasing superoxide dismutase (SOD), glutathione (GSH), and glutathione peroxidase (GPx) levels and decreasing lipid peroxidation (MDA) in spleen tissues, where oxidative stress increased with radiation exposure. In addition, M-AgNPs exert anti-inflammatory effects by increasing the level of the IleSnt information regulator 1 (SIRT1) protein, which plays an essential role in the control of oxidative stress at the molecular level, while decreasing the levels of inflammatory markers induced by increased oxidative stress in spleen tissues [57].

Matcha tea (150 mg/kg body weight) and the selective cyclooxygenase-2 (COX-2) inhibitor etoricoxib (10 mg/kg body weight) were administered to female rats with acute kidney injury induced by ionizing γ radiation, and their anti-inflammatory effects were investigated. Anti-inflammatory effects were observed in both treatment groups. Furthermore, in addition to the anti-inflammatory effect of aqueous matcha tea extract, its healing effect on kidney tissue, attributed to its antioxidant properties, was also determined. Matcha tea effectively mitigates acute kidney damage caused by ionizing radiation, which is used for diagnosis and treatment, likely because of its phenolic composition [58].

Despite the promising antioxidant and anti-inflammatory properties reported for matcha tea, the currently available evidence should be interpreted with caution. Much of this evidence is derived from *in vitro* experiments and animal models employing controlled experimental conditions, specialized matcha formulations, or doses that may not directly reflect typical human consumption patterns. Furthermore, differences in matcha preparation, extraction methods, and food matrices may influence the bioavailability and biological activity of its bioactive compounds. Although preliminary clinical findings support potential health benefits, well-designed human intervention studies remain limited. Therefore, additional clinical investigations are needed to establish dose–response relationships, determine the physiological relevance of the reported mechanisms, and clarify the long-term antioxidant and anti-inflammatory effects of matcha consumption in humans. Beyond its antioxidant properties, matcha tea has also been investigated for its antimicrobial, oral health–promoting, anticancer, and anti-inflammatory activities. The available evidence from *in vitro*, *in vivo*, and clinical studies is summarized in Table 2.

### 6.2. Antimicrobial Activity

Matcha tea (20 mg/mL) exhibited a bactericidal effect against *Streptococcus pneumoniae*, regardless of antibiotic resistance. Additionally, it inhibits pneumolysin toxin, a virulence factor that plays a crucial role in pneumococcal pneumonia [60]. Edraki et al. (2022) [61] reported that matcha tea was effective against Gram-positive and Gram-negative bacteria, as well as *Candida albicans* strains. Nevertheless, it was not effective against the *Aspergillus niger* strain [61]. In a previous study, the aqueous extract of matcha tea was observed to inactivate SARS-CoV-2 Omicron subvariants in 10 s. The observed effect was associated with catechins [59].

The antiamoebic effects of matcha tea brewed in cold and hot water at a concentration of 0.01 g/mL against *Acanthamoeba castellanii* trophozoites and cystic forms were investigated *in vitro*. The infusion of matcha tea, which was prepared in both hot and cold water, demonstrated an antiamoebic effect through the inhibition of trophozoite growth. The temperature of the infusions did not affect the compounds responsible for the antiamoebic activity. The mechanism underlying the antiamoebic effect of matcha tea is associated with increased glycogen and carbohydrate levels in trophozoites and cysts [62].

Current evidence indicates that matcha possesses antimicrobial potential; however, several limitations should be considered when these findings are interpreted. Most of the available evidence is derived from *in vitro* studies employing concentrated matcha extracts or isolated preparations under controlled laboratory conditions that may not reflect the concentrations achievable through typical dietary consumption. Moreover, differences in extraction procedures, microbial species, and experimental protocols contribute to the variability among studies. Therefore, although the reported findings are encouraging, additional well-designed *in vivo* and clinical investigations are needed to establish the practical relevance of these antimicrobial effects in humans. The available evidence from antimicrobial studies of matcha tea is summarized in Table 2.

### 6.3. Oral Health

The effectiveness of an oral care product containing organic matcha tea, plant-derived antimicrobial enzymes, ascorbic acid, dextrose, sodium hydroxide, and water has been reported to reduce plaque formation, prevent gingivitis and tartar formation, and maintain oral freshness. Moreover, no adverse effects were observed in dogs [63]. A clinical study involving 40 patients with gingivitis investigated the effects of consuming matcha tea or green tea twice daily for 1 month on oral health. In this study, green tea resulted in a greater reduction in gingival size than matcha tea. In contrast, patients who consumed matcha tea reported greater comfort, which was attributed to increased salivary flow and its potential antistress effects [67]. Although preliminary clinical findings suggest that matcha tea may contribute to improvements in oral health, the available evidence remains limited by the small number of studies and variations in study design, intervention duration, and matcha formulations. Additional well-designed clinical studies are needed to confirm these findings and determine their long-term clinical significance. The available evidence regarding the effects of matcha tea on oral health is summarized in Table 2.

### 6.4. Anticancer Activity

Matcha tea extracts have been studied for their ability to reduce the viability of breast cancer cells (MCF-7 and MDA-MB-231) and prevent their proliferation. Compared with the ethanol extract, the aqueous extract of matcha tea is more effective in terms of its antiproliferative and cytotoxic properties. Matcha tea increases oxidative stress, and its antiproliferative and cytotoxic effects may be linked to this pro-oxidant effect [65]. At a concentration of 0.2 mg/mL, matcha tea significantly inhibited the spread of cancer stem cells. A previous study revealed that matcha tea has antioxidant and anti-inflammatory effects, disrupting metabolic processes such as mitochondrial respiration and glycolysis and regulating the cell cycle through various pathways [66]. A study examining the effect of matcha tea on PPAR-γ expression in T47D cells, a model of ductal breast cancer, revealed that matcha tea exerts an anticancer effect by increasing PPAR-γ expression and enhancing cell permeability [64]. Additionally, matcha tea extract exhibited cytotoxic effects on WERI-Rb-1 cells, inducing apoptosis and increasing the number of cells in the early phase of division, which can be attributed to its pro-apoptotic effect on WERI-Rb-1 cells, a retinoblastoma cell line [33].

Despite several lines of evidence that suggest promising anticancer potential, important limitations should be considered when these findings are interpreted. Most of the reported anticancer effects have been demonstrated in *in vitro* cell line models using relatively high concentrations of matcha extracts that may not reflect the concentrations of bioactive compounds achievable through typical dietary consumption. Furthermore, cellular responses may vary among cancer types, experimental conditions, and extraction methods, limiting direct comparisons across studies. Although these findings provide valuable mechanistic insights into the biological activity of matcha-derived compounds, additional well-designed animal studies and human clinical trials are needed to determine whether these anticancer effects are reproducible under physiological conditions and are clinically relevant. The available evidence from studies investigating the anticancer activity of matcha tea is summarized in Table 2.

According to a systematic review, the high polyphenol content of matcha tea has been proposed as a potential contributor to antiviral, immunomodulatory, and anticancer activities; however, these proposed benefits require confirmation in well-designed clinical studies before prophylactic use can be recommended for cancer patients or immunocompromised individuals [68].

**Figure 3:**
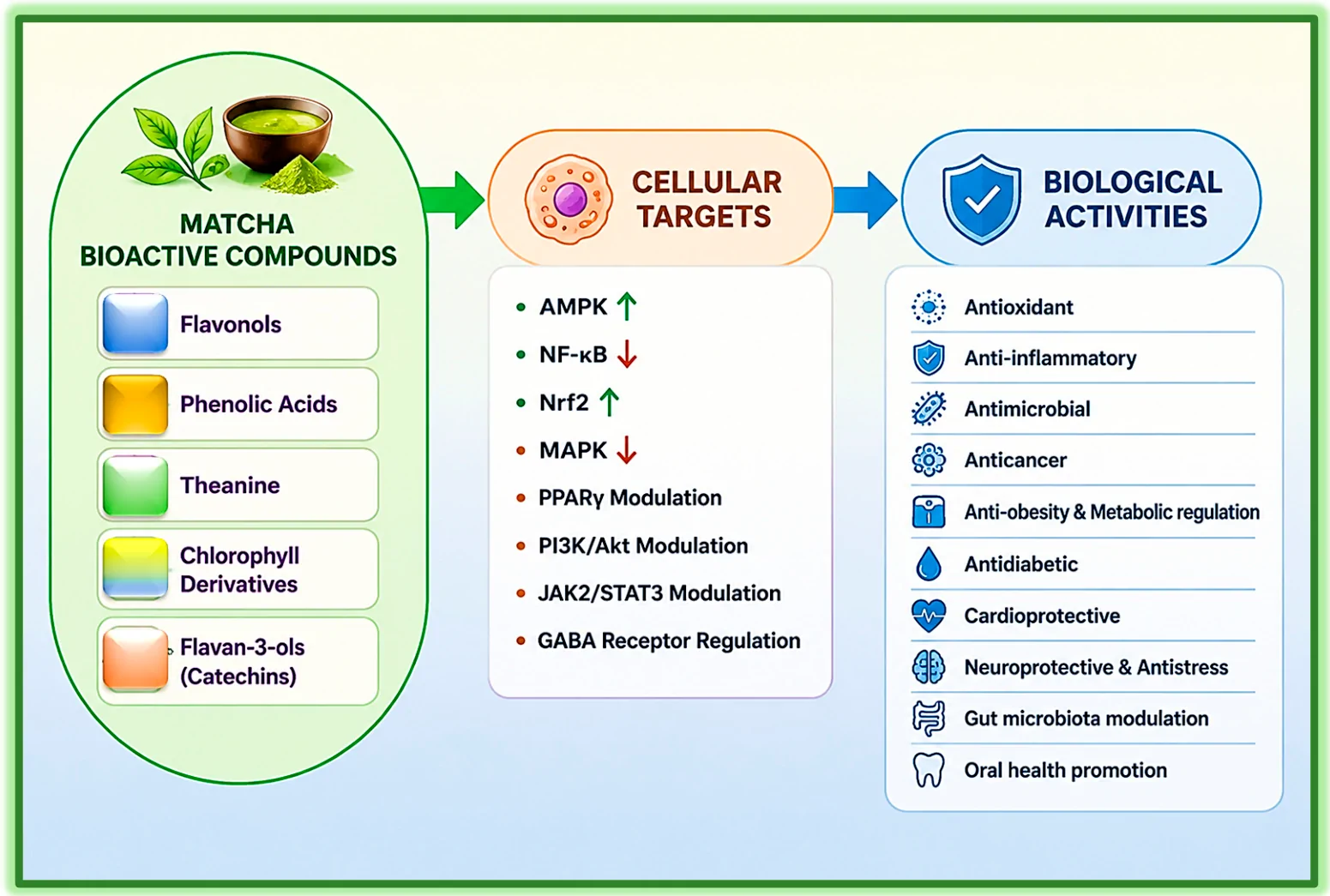
Biological activities of matcha tea and their underlying molecular targets. Matcha tea contains a variety of bioactive compounds, including flavonols, phenolic acids, theanine, chlorophyll derivatives, and flavan-3-ols (catechins), which modulate multiple intracellular signaling pathways. These compounds have been reported to modulate multiple intracellular signaling pathways, such as AMP-activated protein kinase (AMPK), nuclear factor erythroid 2–related factor 2 (Nrf2), nuclear factor kappa B (NF-κB), mitogen-activated protein kinase (MAPK), peroxisome proliferator-activated receptor gamma (PPARγ), phosphoinositide 3-kinase/protein kinase B (PI3K/Akt), Janus kinase/signal transducer and activator of transcription (JAK2/STAT3), and γ-aminobutyric acid (GABA) receptor-associated pathways. Through these molecular mechanisms, experimental studies suggest that matcha tea may have antioxidant, anti-inflammatory, antimicrobial, anticancer, antiobesity, antidiabetic, cardioprotective, neuroprotective, gut microbiota-modulating, and oral health-promoting effects, highlighting its potential as a functional food for supporting metabolic, neurological, cardiovascular, and overall health.

These findings collectively suggest that matcha tea exerts pleiotropic biological effects through the modulation of multiple cellular signaling pathways. Figure 3 schematically illustrates the proposed relationships among matcha bioactive compounds, their molecular targets, and the potential health-promoting activities of matcha tea.

### 6.5. Effects on Weight Control, Metabolism, and Diabetes

The antiobesity effects of matcha tea are linked to its ability to suppress neuroinflammation in the hypothalamus by preventing the secretion of inflammatory cytokines in mice, which tends to increase because of saturated fatty acids [69]. Additionally, a study revealed that a low-quality matcha tea sample rich in catechins strongly inhibited carbohydrate absorption, indicating its potential as a functional food ingredient [37].

Furthermore, a study indicated that consuming matcha tea at doses of 100 and 200 mg/kg over a period of 16 weeks could be beneficial for reducing liver and kidney damage associated with diabetes [70]. The long-term consumption of 2% matcha tea has been shown to have a protective effect on capillaries in mice against aging, with an angiogenic effect linked to vitamin K1 and luteolin [71].

Moreover, matcha tea has been shown to lower blood glucose levels, regulate lipid levels by increasing high-density lipoprotein (HDL) levels, decreasing low-density lipoprotein (LDL) levels, and stimulating endogenous antioxidant activity in mice [72]. The aqueous extract of matcha tea includes caffeine, protein, amino acids, sugars, catechins, and catechin monomers, reducing adipocyte size and the levels of blood sugar, triglycerides, total cholesterol, alanine aminotransferase (ALT), and aspartate aminotransferase (AST) in a dose-dependent manner in obese mice fed a high-fat diet (HFD). matcha tea also decreased the levels of tumor necrosis factor (TNF-α), interleukin 6 (IL-6), and IL-1β, which are implicated in the development of nonalcoholic fatty liver disease (NAFLD). Its mechanism of action against fatty liver tissue has been attributed to its anti-inflammatory effects, which reduce the expression of proteins associated with lipid droplets and increase the activity of cytochrome P450 [73]. Wang et al. reported that matcha tea samples, which are rich in catechins and catechin esters, presented antioxidant properties, reduced fat accumulation, and beneficial effects on obesity and metabolic syndrome via the regulation of lipid and blood glucose profiles [74]. Most of the evidence supporting the antiobesity, antidiabetic, and lipid-lowering effects of matcha tea originates from animal studies employing relatively high doses or long intervention periods. However, the translational relevance of these findings to humans remains uncertain because the administered doses are not directly comparable to typical dietary intake. Clinical evidence remains relatively limited and less consistent than findings from animal models. El-Elimat et al. (2022) [75] conducted a 12-week pilot observational study in overweight and obese individuals and reported reductions in fasting blood glucose, body mass index, insulin, and leptin levels together with increased HDL-C concentrations following matcha consumption. Nevertheless, most of these changes did not significantly differ from those observed in the control group, limiting conclusions regarding the metabolic efficacy of matcha. Furthermore, the relatively modest sample size of this pilot study may limit its statistical power and the generalizability of its findings.

The consumption of four cups of matcha tea within 24 h was found to be associated with increased fat oxidation among women who participated in a moderate exercise regimen [76]. A further clinical study demonstrated that long-term ingestion of matcha tea powder (3 g per day for three weeks) resulted in increased fat oxidation and decreased carbohydrate oxidation. These findings suggest that metabolic adaptations can be achieved through strength exercises [77].

The effects of matcha tea powder (1.5 g) in capsule form on the fecal microbiota of healthy individuals were also investigated in a clinical study and compared with those of a placebo. Although there was no change in fecal formation or defecation frequency after 2 weeks, the consumption of matcha tea increased the abundance of *Coprococcus*, a beneficial bacterium, whereas it decreased the abundance of *Fusobacterium*, a pathogenic bacterium, in the fecal microbiota [78]. A clinical trial revealed that consuming approximately 2 g of matcha tea daily improved the intestinal microbiota in healthy individuals and had a protective effect against atherosclerosis and other cardiovascular system diseases, with positive effects on vascular endothelial function [79]. The effects of 8 and 12 weeks of matcha tea consumption (1.5 g) on the ability of muscles to adapt to resistance exercises were investigated in a clinical study with young men. The consumption of matcha tea for 8 weeks increased leg strength. Muscle weight has been reported to increase in response to resistance exercise, stimulating muscle adaptation during resistance exercises, reducing postexercise fatigue and stress, and contributing to the adaptation provided by exercise, along with the postexercise recovery process [80]. However, a study in hypercholesterolemic rabbits revealed that long-term consumption of matcha tea impaired reverse cholesterol transport, increased arteriosclerosis, and increased susceptibility to the development of atherosclerotic lesions [81]. Collectively, the available evidence suggests that matcha tea may positively influence metabolic health through the modulation of glucose metabolism, lipid homeostasis, inflammatory responses, and substrate oxidation. However, these effects are supported predominantly by animal studies employing relatively high doses of matcha extracts or prolonged interventions. In contrast, relatively few human studies exist and are generally characterized by modest sample sizes, short intervention periods, and mixed findings, with some reported metabolic improvements failing to differ significantly from control interventions. Although the available clinical studies employed appropriate randomized or controlled intervention designs commonly used in pilot nutritional investigations, their limited statistical power may restrict the detection of modest metabolic effects. Consequently, the current clinical evidence is insufficient to establish definitive recommendations regarding matcha consumption for weight management or diabetes prevention. Larger, adequately powered randomized controlled trials using standardized matcha preparations and longer follow-up periods are warranted to determine the clinical relevance and reproducibility of the promising findings observed in preclinical models.

The effects of matcha tea on the gut microbiota, diabetes, and metabolic parameters are shown in Table 3.

### 6.6. Effects on the Central Nervous System

Matcha tea and caffeine-free matcha tea have been shown to improve aging and cognitive disorders by regulating the expression of hippocampal proteins associated with these conditions in mice [82]. In another study investigating the effects of caffeine-containing and decaffeinated matcha tea samples on stress and anxiety-like behaviors in mice, the adrenal glands were shown to grow with the activation of the hypothalamic-pituitary-adrenal (HPA) axis associated with stress. In contrast, matcha tea had an antistress effect that did not affect HPA axis activation. In addition, the mechanism by which continuous consumption of matcha tea reduces anxiety-like behaviors may involve caffeine in matcha tea, increasing the expression of GABA type A receptors [10]. Matcha tea activated the dopaminergic system and had an antidepressant effect on mice that developed depression accompanied by social isolation [55]. The optokinetic responses and visual function effects of sencha green tea, matcha tea, and EGCG were also investigated in young and aged mice. This study revealed that matcha tea increased the temporal sensitivity of optokinetic responses and that both green tea and EGCG could prevent the decline in visual function caused by aging [83]. The effects of aqueous matcha tea extract on cognitive disorders and neurotoxicity were investigated in mice exposed to air pollutants with particle sizes less than 2.5 μm (PM2.5). Aqueous matcha tea extract regulates endogenous antioxidant systems in the respiratory tract, brain, and skin, which are the exposure routes of substances lower than PM2.5, regulating acetylcholine content. Aqueous matcha tea extract is considered a crucial functional food ingredient for mitigating inflammation and neurotoxic effects caused by air pollution, particularly because of its anti-inflammatory properties [84].

Moreover, a clinical study [85] demonstrated that the use of matcha tea (2 g in capsule form) for 2 weeks has a positive effect on mild acute stress and improves cognitive function in young adults under stress. The effects of consuming biscuits containing 4.5 g of matcha tea powder on stress were investigated for 15 days. Matcha tea paired with sweet products exhibited an antistress effect when the ratio of caffeine to EGCG and theanine to arginine was two or less. The antistress effect of matcha tea was found to depend on the ratio of caffeine to EGCG and theanine to arginine [86]. A clinical study of elderly women revealed that consuming matcha tea for 12 weeks improved cognitive function. This healing effect was attributed to the fact that matcha tea eliminated vitamin K deficiency in those who were deficient in vitamin K. In contrast, the improvement in cognitive functions observed in the presence of vitamin E deficiency was explained by the combined effect of vitamin E and polyphenolic compounds [14]. In addition, the effects of consuming matcha tea in its powder form and incorporating it into a fruit bar on cognitive function were investigated in another clinical study.

**Figure 4:**
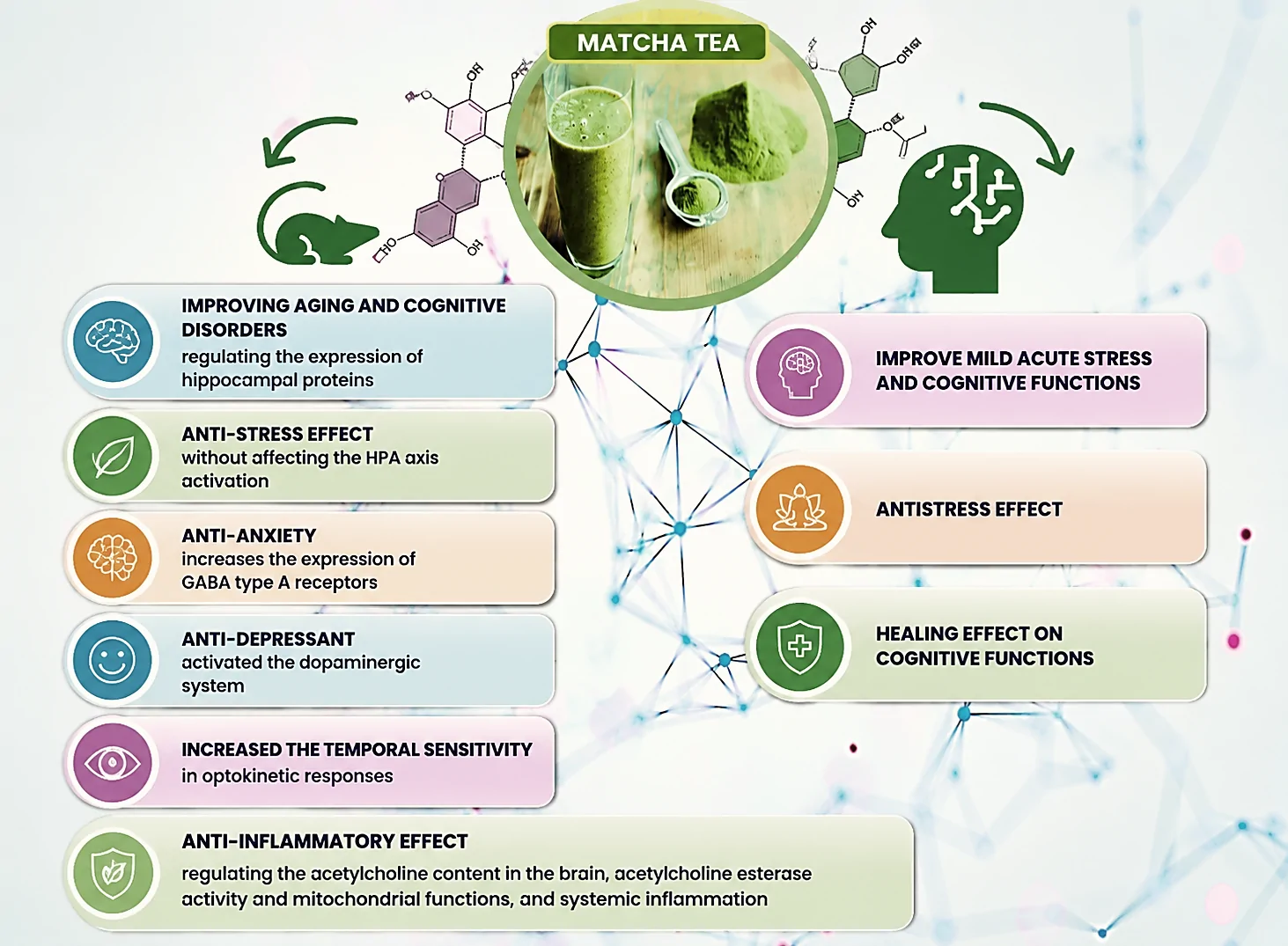
Mechanisms of the central nervous system (CNS) of matcha tea in mice and humans.

Matcha tea in both forms did not change mood. However, similar to the psychoactive effect of caffeine alone, theanine alone was less effective, and a synergistic effect of theanine and caffeine was also observed. In brief, in this specific study evaluating the effects of matcha tea on mood and cognitive performance, the effects of matcha tea powder (4 g) in both product formats (liquid and solid) on mood and cognitive performance were examined. Mood was not significantly affected in either format. Both matcha tea formats had the most consistent effect on attention skills, above all, on working memory [87]. Therefore, the available evidence suggests that matcha tea may exert neuroprotective, cognition-supporting, and antistressing effects through the modulation of neurotransmitter signaling, oxidative stress, and inflammatory pathways. However, the majority of these findings originate from animal studies employing relatively high doses of matcha preparations or purified components that may not accurately reflect typical human dietary consumption patterns. Human intervention studies remain relatively scarce and are generally characterized by short intervention periods, modest sample sizes, and heterogeneous study methodologies, which limit the generalizability of their findings. Moreover, some reported cognitive benefits may be influenced by the nutritional status of study participants, as improvements observed in individuals with vitamin K deficiency may partially reflect the correction of underlying nutritional inadequacies rather than the direct effects of matcha itself. Consequently, current clinical evidence remains insufficient to establish definitive conclusions regarding the neuroprotective efficacy of matcha tea in humans. Larger, adequately powered randomized controlled trials employing standardized matcha preparations and longer follow-up periods are needed to clarify both the magnitude and mechanisms of these potential neurological benefits. The neuroprotective effect of matcha tea, along with its expected mechanisms, is shown in Figure 4.

### 6.7. Processing-Induced Mechanistic Shifts: From Chemistry to Pathway Modulation

In addition to their antioxidant and anti-inflammatory effects, processing-induced transformations of matcha phytochemicals can alter their ability to modulate molecular signaling pathways. For example, thermal epimerization of (−)-epigallocatechin-3-gallate (EGCG) into gallocatechin gallate (GCG) preserves radical-scavenging activity but reduces the ability of EGCG to activate AMP-activated protein kinase (AMPK) and regulate peroxisome proliferator-activated receptor gamma (PPARγ). This attenuation diminishes the efficacy of metabolic regulation and glucose–lipid homeostasis, underscoring that antioxidant equivalence does not translate into functional equivalence [88]. Similarly, the degradation of chlorophyll into pheophytins and pyropheophytins under hot and acidic conditions not only decreases color but also weakens the ability of chlorophyll to bind mutagens and influence aryl hydrocarbon receptor (AhR) signaling. These changes reduce anticancer potential, highlighting that pigment stability has mechanistic implications beyond sensory quality [88]. Theanine, a key amino acid in matcha, is relatively stable but can be degraded under prolonged heat or can form complexes with proteins. Such transformations limit its bioavailability and reduce its ability to cross the blood–brain barrier, thereby attenuating its role in modulating glutamatergic signaling and enhancing *γ*-aminobutyric acid (GABA) activity. Compared with traditional tea infusions, the neuroprotective and antistress effects are diminished in processed food applications [89].

## 7. Matcha in Food Matrices: Processing Stability, Sensory Trade-Offs, and Bioaccessibility

When incorporated into foods, matcha is exposed to processing conditions and matrix interactions that fundamentally differ from those of traditional tea preparation, necessitating a distinct evaluation of its bioactive stability, transformation, and functionality. The effects of incorporating matcha into foods can be understood through a processing–transformation–function framework in which thermal and matrix-related stresses induce chemical changes that, in turn, modify functional efficacy, sensory quality, and safety outcomes.

Within this framework, several studies have examined the incorporation of matcha into starch-based and fermented food systems to assess its technological and nutritional effects. For example, investigations on matcha-enriched rice noodles have demonstrated that the addition of matcha influences starch digestibility and the glycemic response while simultaneously improving textural attributes such as chewiness and elasticity, as well as sensory properties such as color and aroma. However, these benefits are concentration dependent, with optimal quality typically observed at inclusion levels of approximately 1%, whereas higher additions may disrupt the three-dimensional network structure of the food matrix [90].

Similarly, the incorporation of matcha into rice cakes increased the resistant starch content and antioxidant capacity while inhibiting *in vitro* starch digestion, resulting in a reduced glycemic index [91]. These effects can be attributed in part to catechins, which may mitigate oxidative stress through interactions with reactive carbonyl compounds generated during baking [92]. In baked products such as biscuits, thermal processing at approximately 180 °C has been shown to reduce the catechin content by approximately 20%, decrease caffeine levels by approximately 24%, and promote catechin epimerization, highlighting the sensitivity of matcha bioactives to high-temperature processing [93]. Additional studies have reported the effects of the incorporation of matcha on fermented dairy products, including the modulation of acidity and consistency during storage [94], as well as high fluoride retention when it is consumed as a food ingredient or beverage [95].

### 7.1. Stability and Degradation of Catechins in Matcha-Containing Foods

The catechins of matcha tea incorporated into food products are subjected to thermal processing, oxidative conditions, and interactions with starch and protein matrices that differ markedly from those of traditional tea preparations [96]. Studies on matcha-enriched rice noodles and rice cakes have indicated that processing can reduce the total catechin content while simultaneously modulating starch digestibility and the glycemic response. In baked products such as biscuits, thermal treatment at approximately 180 °C resulted in an approximately 20% reduction in catechin content, highlighting the sensitivity of gallated catechins to prolonged heat exposure.

Catechins represent the dominant polyphenolic fraction of matcha tea; however, their stability is highly dependent on processing conditions. When matcha is incorporated into solid foods such as biscuits, noodles, or baked products, catechins are exposed to prolonged thermal treatment and oxidative environments that differ markedly from those of aqueous infusion. Several studies indicate that baking temperatures exceeding 160–180 °C result in substantial reductions in total catechin content, with losses typically ranging from 15% to 30%, depending on time and formulation. The extent of degradation is catechin specific. Compared with nongallated catechins such as epigallocatechin (EGC), gallated catechins such as epigallocatechin gallate (EGCG) are more susceptible to thermal degradation and oxidation during processing [97]. However, processing-induced transformations do not necessarily represent a complete loss of functionality, as the resulting epimeric forms may retain biologically relevant, albeit altered, bioactive properties [97]. In starch-rich matrices, interactions between catechins and amylose or amylopectin may partially protect these compounds from oxidative degradation, whereas in low-moisture baked products, oxygen diffusion accelerates catechin loss. Additionally, the presence of lipids and proteins can modulate catechin stability through the formation of noncovalent complexes, which may reduce immediate degradation but also alter release during digestion [98]. Overall, while incorporation into food matrices reduces the absolute catechin content, matrix-dependent protective effects may preserve specific catechin fractions and influence their physiological relevance.

### 7.2. Heat-Induced Epimerization of Catechins During Food Processing

In addition to degradation, thermal processing induces the epimerization of catechins, a transformation that has been largely overlooked in previous reviews by matcha. Epimerization involves the conversion of thermolabile catechins, such as EGCG and EGC, into their more heat-stable epimers, gallocatechin gallate (GCG) and gallocatechin (GC), respectively. This process occurs primarily at elevated temperatures and extended heating times, such as those encountered during baking or extrusion.

Although epimerized catechins retain their antioxidant capacity, emerging evidence suggests that their bioactivity in metabolic regulation and anticancer pathways may differ from that of their native counterparts. Consequently, foods containing processed matcha may exhibit preserved antioxidant effects while showing altered biological efficacy in other functional domains. This distinction is particularly relevant when evaluating the health claims of matcha-enriched foods, as the total polyphenol content alone does not reflect qualitative shifts in the catechin composition [12].

### 7.3. Caffeine Retention and Exposure from Matcha-Enriched Foods

Caffeine is a thermally stable alkaloid and is therefore largely retained during food processing. Unlike catechins, caffeine undergoes minimal degradation during baking, drying, and storage, contributing to its high stability in matcha-enriched foods [15]. The caffeine content of matcha powder varies considerably according to cultivar, shading practices, harvest time, and product quality and is consequently reflected in the final caffeine concentration of matcha-containing food products [15,17]. Studies investigating the incorporation of matcha into baked foods have demonstrated that caffeine remains detectable after thermal processing, supporting its considerable thermal resilience under conventional manufacturing conditions [93].

Unlike matcha beverages, where caffeine intake is influenced by brewing parameters and dilution, matcha-containing foods may provide caffeine in a more concentrated and less perceptible form. Consequently, cumulative caffeine exposure may occur when multiple matcha-enriched products are consumed concurrently or alongside other sources of dietary caffeine. However, the actual risk of excessive caffeine intake depends largely on product formulation, service size, and consumption frequency. Consumption of a single matcha-containing food product is unlikely to pose concerns for most healthy adults, whereas repeated consumption of highly fortified products may substantially increase total caffeine exposure. Sensitive populations, including children, pregnant women, and individuals with caffeine sensitivity, may be particularly susceptible to adverse effects at lower intake levels [15].

Therefore, caffeine retention should be considered not only as a functional attribute contributing to stimulant effects but also as a safety parameter in the formulation and labeling of matcha-based food products. Future studies should establish standardized caffeine profiles for commercially available matcha-enriched foods and evaluate cumulative dietary exposure under realistic consumption scenarios.

### 7.4. Stability of L-Theanine and Functional Balance with Caffeine

L-theanine (*γ*-glutamylethylamide) is the predominant nonproteinogenic amino acid in matcha tea and plays a central role in its antistress and cognitive effects, primarily through its synergistic interaction with caffeine. However, compared with caffeine, L-theanine is far more susceptible to thermal degradation. While caffeine exhibits high thermal resilience across standard processing routines (>95% retention), the open-chain structure of L-theanine makes it vulnerable to thermooxidative degradation, thermal cleavage into L-glutamic acid and ethylamine, and nonenzymatic browning (Maillard reactions with reducing sugars) when it is processed in complex or solid food matrices [99,100]. Furthermore, L-theanine can interact with food proteins—particularly in dairy-based matrices—potentially altering its extraction efficiency and bioaccessibility.

Quantitative studies indicate that while standard aqueous infusions at moderate temperatures (<80 °C for short durations) retain more than 98% of the initial L-theanine, severe thermal processing causes significant, quantifiable losses [101]. Specifically, ultrahigh-temperature (UHT) beverage sterilization, extended hot-fill holding, and matrix baking (160–180 °C) induce L-theanine reductions ranging from 15% to 35%, with degradation accelerating in acidic liquid environments (pH < 4.0). Because processing selectively degrades L-theanine while preserving caffeine, it systematically increases the caffeine-to-L-theanine ratio.

This processing-induced shift may alter the functional equilibrium that contributes to the psychophysiological benefits of matcha. According to the CE/TA molar ratio model proposed by Unno et al. (2019) [86], the stress-reducing effects of matcha are optimized when the combined molar ratio of caffeine and epigallocatechin gallate (EGCG) relative to L-theanine and arginine remains below 2.0. Exceeding this threshold diminishes the antistress effects observed in experimental models, suggesting that the relative proportions of these bioactive compounds are functionally important [86]. Moreover, clinical evidence has indicated that the combined administration of L-theanine and caffeine enhances selective attention and cognitive performance more effectively than either component alone does, underscoring the importance of their synergistic interaction [99]. Consequently, preferential losses of L-theanine during severe thermal processing or reduced bioaccessibility arising from food matrix interactions could theoretically shift the functional profile of matcha toward greater stimulant predominance by weakening the modulatory influence of L-theanine on caffeine-induced neural responses [86,99]. However, direct evidence demonstrating that processing-induced changes in the caffeine-to-L-theanine ratio translate to altered cognitive or antistress outcomes in humans remains limited. Future studies should therefore quantify these changes across different matcha-containing food matrices and determine their physiological significance.

### 7.5. Chlorophyll Degradation, Color Stability, and Consumer Acceptance

The characteristic bright green color of matcha is attributed primarily to its high chlorophyll content and is a critical determinant of consumer perception and product acceptability. Chlorophyll stability is influenced by several processing- and formulation-related factors that collectively determine color retention and consumer acceptance in matcha-containing foods [88,102]. The major factors affecting chlorophyll degradation, color stability, and consumer acceptance are described below.

(1)  Temperature and thermal processing

Chlorophyll is highly susceptible to thermal degradation during food processing. High temperatures encountered during baking and prolonged heat treatments accelerate the conversion of chlorophyll into pheophytins and pyropheophytins, resulting in color dulling and undesirable brown hues. Consequently, thermal processing conditions must be carefully optimized to preserve the characteristic green color of matcha-containing products.

(2)  Acidic conditions (pH)

Acidic food matrices, such as fermented dairy products and yogurts, promote chlorophyll degradation by facilitating its conversion into pheophytins. This pH-dependent transformation compromises color stability and adversely affects the visual quality of matcha-enriched foods, thereby influencing consumer acceptance.

(3)  Oxygen exposure

Exposure to oxygen during processing and storage contributes to chlorophyll oxidation and pigment degradation. Oxidative changes further diminish the intensity of green coloration and may negatively affect the overall appearance and quality of matcha-containing products. Therefore, appropriate packaging and storage conditions are important for maintaining pigment stability.

(4)  Matcha inclusion level

The concentration of matcha powder incorporated into food products significantly influences both functional and sensory attributes. High inclusion levels increase the antioxidant potential and may increase the nutritional value of the final product. However, excessive amounts of matcha can intensify bitterness and produce undesirable color changes, thereby limiting consumer acceptability. Consequently, determining the optimal inclusion level is essential for balancing functional benefits with sensory quality.

(5)  Sensory attributes and consumer acceptance

Consumer acceptance of matcha-enriched foods is strongly influenced by visual appearance, color intensity, and flavor characteristics. Products exhibiting substantial chlorophyll degradation often display dull or brownish coloration and increased bitterness, which negatively affect sensory perception despite their potential health benefits. Therefore, sensory quality should be considered along with the preservation of bioactive compounds during product formulation.

(6)  Quantitative aspects of chlorophyll degradation and preservation strategies

The extent of chlorophyll degradation is highly dependent on the processing conditions. During tencha processing, the concentration of chlorophyll decreased from 3.45 mg/g in fresh leaves to 2.41 mg/g after fixation and 2.14 mg/g after drying, corresponding to losses of approximately 30.1% and 38.0%, respectively [38]. The susceptibility of chlorophyll b to degradation increased, decreasing from 1.66 mg/g to 0.62 mg/g after fixation and to 0.56 mg/g after drying, representing losses of 62.7% and 66.3%, respectively. Thermal processing promotes the conversion of chlorophylls into pheophytins and related degradation products, resulting in the progressive loss of the characteristic bright green color of plant-derived foods [103,104]. Moreover, oxygen, light exposure, and storage conditions contribute substantially to pigment oxidation and color deterioration [105]. Consequently, preserving the characteristic green color of matcha-containing foods may be facilitated by minimizing thermal exposure through lower processing temperatures and shorter heating times, limiting oxygen and light exposure during storage, and optimizing formulation and processing conditions whenever technologically feasible.

Collectively, these factors influence chlorophyll degradation, color stability, and consumer acceptance of matcha-containing foods. Optimizing processing conditions, formulation parameters, and storage practices is therefore necessary to balance color retention, functional efficacy, and sensory quality while maximizing the consumer acceptability of matcha-enriched products [88,102].

### 7.6. Digestive Bioaccessibility of Matcha Bioactives in Food Matrices

Bioaccessibility, defined as the fraction of a compound released from the food matrix during digestion and available for absorption, is a critical determinant of the physiological impact of matcha bioactives. *In vitro* digestion studies have indicated that although thermal processing reduces the total polyphenol content, its incorporation into solid food matrices may increase gastrointestinal stability by protecting catechins from premature degradation in the upper digestive tract [106]. A substantial proportion of matcha phenolics remain nondigestible and reach the colon, where they may exert prebiotic-like effects through interactions with the gut microbiota. This delayed release contrasts with the rapid absorption observed for tea infusions and suggests that matcha-enriched foods may provide sustained bioactive delivery rather than immediate systemic exposure [38]. Consequently, the health implications of matcha consumption differ markedly between beverage and food formats, emphasizing the need to consider matrix-dependent bioaccessibility when evaluating functional outcomes [107]. Together, these processing- and matrix-dependent transformations underscore the importance of evaluating matcha not only as a phytochemical source but also as a complex food ingredient whose functional, sensory, and safety attributes are intrinsically linked.

## 8. Matcha Beyond the Cup: Integration into Food Systems

While matcha has traditionally been consumed as a beverage, its growing use as a functional ingredient in diverse food systems requires a systematic evaluation of how processing and matrix interactions alter its stability, release kinetics, and sensory properties. Unlike tea infusions, where bioactives are extracted into water, matcha incorporated into bakery, dairy, confectionery, and nutraceutical products is subjected to complex physicochemical environments that fundamentally reshape its functional profile. Following *in vitro* gastrointestinal digestion, matcha exhibited digestibility values ranging from 61.2% to 65.8%, whereas epigallocatechin gallate (EGCG) and epigallocatechin (EGC) were among the most readily released phenolics, with residual levels less than 1%, indicating relatively high digestive release compared with less bioaccessible compounds such as rutin and quercetin [26]. In contrast, protein-rich food matrices can substantially reduce the bioavailability of galloylated catechins, while interactions with dairy components and digestive enzymes further modulate catechin release and antioxidant activity during digestion [108,109,110,111]. These observations highlight that the practical functionality of matcha-containing foods is determined not only by the initial phytochemical composition but also by matrix-dependent effects on bioaccessibility during digestion.

### 8.1. Bakery Products

Thermal processing at high temperatures (≥180 °C) promotes catechin epimerization (EGCG → GCG) and reduces the overall catechin content, with kinetic studies showing increased degradation and formation of epimers as the temperature increases [112]. Starch-rich matrices can partially protect catechins by limiting oxygen diffusion and forming polyphenol–starch complexes, yet low-moisture baked goods accelerate degradation and limit extractability, altering release and absorption profiles [113]. Sensory masking is achieved through sugar and fat, which reduce bitterness, whereas theanine contributes to umami balance. Importantly, compared with traditional tea infusions, catechins bound to starch or proteins may exhibit delayed release during digestion, thereby altering their bioaccessibility [113].

### 8.2. Dairy Systems

In dairy matrices, proteins such as casein and whey form noncovalent complexes with catechins, stabilizing them against oxidation but reducing their immediate bioavailability, as evidenced by reduced plasma catechin levels when catechins are consumed with milk proteins [108]. Moreover, catechins can associate with protein and milk fat globules, indicating that both the protein and lipid components of dairy products influence polyphenol behavior in food systems [111]. During digestion, the presence of dairy matrices has been shown to improve polyphenol stability and maintain antioxidant activity relative to those of control systems, suggesting protective effects in the gastrointestinal tract [109]. Competitive interactions between catechins and milk proteins also modulate *in vitro* bioaccessibility and antioxidant potential, with differential effects depending on the specific protein and digestion conditions [110]. Sensory masking is particularly effective in dairy products, where protein–polyphenol interactions can reduce astringency and bitterness, increasing consumer acceptability. Matcha-enriched yogurt and kefir thus represent promising vehicles for functional delivery.

### 8.3. Confectionery and Chocolate

Fat-rich confectionery systems can stabilize catechins during storage by limiting oxidative stress and providing a protected lipid environment for polyphenols, as shown in lipid-based encapsulation studies utilizing cocoa butter matrices [114]. Compared with aqueous systems, cocoa and other lipid matrices also modify the bioavailability of catechins, likely by delaying their release and altering their absorption sites [115]. Moreover, encapsulation strategies such as inclusion complexes have been shown to mask bitterness and improve catechin stability in solid food matrices, supporting improved sensory profiles in functional confections [116]. Cocoa butter itself provides a stable, solid lipid platform commonly used in chocolates and related products, further facilitating the integration of bioactive compounds into palatable formats.

### 8.4. Nutraceuticals and Functional Foods

Encapsulation technologies (liposomes, starch granules, and nanoparticles) are being increasingly applied to matcha bioactives to improve stability and control release. Unlike food matrices, nutraceutical formulations avoid heat degradation but face challenges in terms of digestive bioaccessibility. Optimizing catechin–theanine ratios in capsule or powder forms allows targeted health claims, such as stress reduction or metabolic regulation. This position positions matcha as a versatile ingredient in functional food design beyond traditional beverage formats [117].

## 9. Multiomics and Chemometric Perspectives on Matcha

Multiomics technologies and chemometric approaches provide valuable tools for elucidating the compositional characteristics, biological activities, functional efficacy, and safety profile of matcha tea and matcha-enriched food products. Metabolomic profiling offers critical insights into the *in vivo* transformation of matcha bioactives, particularly when they are consumed within food matrices rather than as traditional infusions. Processing-induced chemical modifications, including catechin epimerization (e.g., EGCG → GCG) and chlorophyll degradation to pheophytins, generate distinct metabolic signatures detectable in plasma and urine that influence antioxidant and metabolic activities, thereby enabling the identification of processing-dependent fingerprints that link chemical transformations to functional outcomes [16]. Furthermore, *in vitro* digestion studies have demonstrated that the food matrix substantially influences catechin bioaccessibility and antioxidant activity, highlighting the importance of considering metabolite profiles after gastrointestinal transformation rather than relying solely on the native phytochemical composition [26,27,113]. Proteomic analyses further revealed how metabolic biomarkers modulate protein expression in key tissues, including the liver, adipose tissue, and brain, through pathways involving AMPK, NF-κB, and PPARγ signaling. Animal studies have additionally demonstrated that matcha consumption influences proteins involved in inflammatory, metabolic, and neuroprotective pathways, supporting the utility of proteomics for identifying molecular targets associated with matcha bioactivity [69,73,82]. However, food matrix interactions, such as protein binding in dairy systems, may attenuate these biological effects, emphasizing the importance of the matrix context when evaluating the functional efficacy of native and processed matcha-containing foods [109,110,111,118]. The gut microbiota also plays a pivotal role in mediating the health effects of matcha constituents, as catechins and theanine influence microbial composition, short-chain fatty acid production, and bile acid metabolism, whereas epimerized catechins may exhibit altered availability to colonic bacteria, thereby affecting fermentation profiles and host metabolic outcomes. Recent human studies have further demonstrated that matcha consumption can induce measurable alterations in gut microbial composition, suggesting that microbiome profiling may provide useful signatures for evaluating individual biological responses to matcha intake and its interactions with food matrices [78,79]. Consequently, microbiome analyses have positioned matcha as a potential prebiotic-like modulator whose bioactivity is substantially influenced by food processing and gastrointestinal metabolism [74]. In conjunction with these approaches, chemometric methods, including principal component analysis (PCA) and partial least squares discriminant analysis (PLS-DA), facilitate the discrimination of tea varieties and product categories on the basis of compositional fingerprints and have been successfully applied for food authentication and quality assessment in tea and other food systems [17,23,119,120,121]. The integration of multiomics datasets further provides a systems-biology framework that links processing conditions with biological responses and health-related outcomes, positioning matcha as a model ingredient for systems-level functional food research [122]. Nevertheless, currently available studies have largely focused on pathway-level biological responses rather than the identification of quantitative metabolomic or proteomic biomarkers that can reliably distinguish matcha products according to processing conditions and food matrices. Consequently, the matrix- and processing-specific omics signatures of matcha remain insufficiently characterized. Future investigations integrating standardized processing models with metabolomic, proteomic, and microbiome analyses will be essential for identifying processing- and matrix-dependent biomarkers predictive of bioaccessibility, functionality, and health outcomes in matcha-enriched foods. Although matcha tea is generally regarded as safe, omics-based approaches offer important opportunities for assessing toxicity and evaluating processing-dependent safety considerations. Evidence from green tea indicates that excessive polyphenol intake may induce oxidative stress, mitochondrial dysfunction, and hepatotoxicity, whereas excessive caffeine consumption may contribute to osteoporosis and hypertension [123]. Furthermore, matcha supplies relatively high levels of fluoride that may approach the recommended daily intake with regular consumption [95], and experimental studies have demonstrated that lyophilized matcha extracts can induce apoptosis in endothelial cells and exhibit toxic effects in plant models such as *Artemisia annua* [13]. However, these reported adverse effects are primarily associated with excessive exposure or experimental conditions and are not necessarily representative of customary dietary consumption patterns of matcha tea. When consumed in traditional serving sizes, matcha is generally considered a safe beverage for healthy adults; nevertheless, cumulative caffeine and fluoride intake may warrant consideration among habitual consumers and susceptible people. Given that the concentrations of bioactive compounds can vary substantially among commercial products, formulations, and serving sizes, additional dose–response investigations and long-term clinical studies are needed to establish evidence-based intake recommendations for matcha- and matcha-enriched foods [124]. Collectively, multiomics and chemometric strategies provide a comprehensive and integrative framework for understanding how composition, processing, metabolism, functionality, and safety collectively determine the health-promoting potential of matcha tea and matcha-enriched foods.

## 10. Conclusions

Matcha tea has emerged as a distinctive form of green tea and a versatile food ingredient owing to its high content of bioactive phytochemicals, including catechins, caffeine, L-theanine, chlorophylls, and essential micronutrients. Differences in cultivation practices, shading, harvesting, and powder-based consumption distinguish matcha from other green teas and contribute to its unique compositional and functional profile. Previous studies have associated the consumption of matcha with a range of biological activities, including antioxidant, anti-inflammatory, antimicrobial, and metabolic effects, although much of the current evidence is derived from *in vitro* and preclinical models.

This review highlights that the health-promoting potential of matcha cannot be predicted solely from its native phytochemical composition but must instead be considered within a processing–transformation–bioaccessibility framework. Processing conditions and food matrix interactions determine not only the stability of matcha bioactives but also their chemical transformations, gastrointestinal availability, and ultimately their biological efficacy. Thermal treatment, formulation parameters, storage conditions, and matrix interactions collectively determine whether matcha bioactives are degraded, chemically transformed, or protected during food processing. Importantly, these changes do not necessarily result in a simple loss of functionality but may alter the balance between antioxidant activity, metabolic regulation, sensory quality, and safety. For example, catechin epimerization may preserve antioxidant capacity while modifying pathway-specific bioactivities, whereas starch and protein matrices may simultaneously attenuate bioactive losses and influence digestive release and bioaccessibility. Consequently, processing-dependent transformations give rise to measurable trade-offs between health-related functionality, sensory quality, and safety, particularly in matcha-enriched baked, fermented, and starch-based products. Recognizing these processing-dependent trade-offs is essential for the rational development of matcha-containing foods and for ensuring that health-oriented product claims are aligned with realistic formulation and manufacturing outcomes. While incorporation into food matrices may attenuate certain bioactive compounds, it may also confer protective effects, modulate glycemic responses, and enable sustained gastrointestinal delivery of phenolics, thereby differentiating food-based matcha consumption from traditional tea infusions. Future research should prioritize well-designed *in vivo* and clinical studies that account for processing history, food matrix effects, and realistic consumption patterns. Greater attention should also be directed toward elucidating the gastrointestinal fate and bioaccessibility of processing-induced transformation products, including epimerized catechins and chlorophyll degradation compounds. The integration of multiomics approaches with standardized processing models may further clarify how food matrices influence molecular targets and health outcomes. Additionally, long-term safety assessments considering cumulative caffeine exposure, fluoride intake, and habitual consumption patterns remain limited and warrant further investigation across different population groups. Addressing these challenges will be essential for translating the growing interest in matcha tea into scientifically substantiated, safe, and effective functional food applications.

## Figures and Tables

**Table 1:** Comparative scope of recent reviews of matcha tea and the unique contributions of the present review.

Review (Year)	Primary Focus	PSB ^1^	FMI ^2^	CDE ^3^	CTFB ^4^	SCTo ^5^	DBF ^6^	SC ^7^
Kochman et al. (2020) [16]	Composition and health effects	❌	❌	❌	❌	❌	❌	⚠ Limited
Sokary et al. (2023) [18]	Therapeutic potential (human and animal studies)	❌	❌	❌	⚠ Beverage-focused	❌	⚠ Infusion-based	⚠ Limited
Phuah et al. (2023) [17]	Origin, processing, composition, and omics	⚠ General processing	❌	❌	❌	❌	❌	⚠ Limited
Kika et al. (2024) [15]	Updated nutrient profiling	❌	❌	❌	❌	❌	❌	❌
Present Review	Matcha in food systems	✔ Detailed	✔ Systematic	✔ Emphasized	✔ Ratio-based evaluation	✔ Processing–acceptance trade-offs	✔ Food matrix-dependent	✔ Integrated and applied

^1^ PSB: Processing stability of bioactive compounds. ^2^ FMI: food matrix interactions. ^3^ CDE: catechin degradation & epimerization. ^4^ CTFB: caffeine and theanine functional balance. ^5^ SCTo: sensory and color trade-offs. ^6^ DBF: digestive bioaccessibility in foods. ^7^ SC: safety considerations (caffeine, fluoride). **Note:** ✔ indicates the criterion is fulfilled; ❌ indicates the criterion is not fulfilled; ⚠ indicates limited fulfillment.

**Table 2:** Effects of matcha tea on antimicrobial, oral health, and anticancer potential.

Study Design	Types of Matcha Tea Forms	Main Results	References
*in vivo*	Silver particles	In rats, SOD, GSH, and GPx enzyme levels ↑, lipid peroxidation ↓, and SIRT1 levels ↑	Hamed et al., 2023 [57]
*in vivo*	150 mg/kg body/weight matcha tea +10 mg/kg body/weight Etoricoxib	Matcha tea and the COX-2 inhibitor Etoricoxib for inflammation ↓. Healing of kidney tissues ↑	Khateeb and Taha 2024 [58]
*in vitro*	Aqueous extract of matcha tea	Inactivated in 10 s in the Omicron subvariants of the SARS-CoV-2 virus	Shin-Ya et al., 2023 [59]
*in vitro*	The supernatant of the matcha tea aqueous extract	Bactericidal and antitoxin effects against antibiotic-susceptible and -resistant *Streptococcus pneumoniae* strains	Sasagawa et al. 2021 [60]
*in vitro*	Matcha tea	Antimicrobial effect (*Bacillus subtilis*, *Staphylococcus aureus*, *S. epidermidis*, *Streptococcus pyogenes*, *Escherichia coli*, *Pseudomonas aeruginosa*, *Salmonella paratyphi*-A serotype, *Shigella dysenteriae*, and *Candida albicans*)	Edraki et al., 2022 [61]
*in vitro*	Aqueous extract of matcha tea	Anti-amoebic (*Acanthamoeba castellanii* by inhibiting the growth of trophozoites)	Dickson et al., 2020 [62]
clinical trial	Oral care product containing matcha tea	28-day study, the significance of a ↓ plaque index and plaque thickness (in dogs)	Lindinger 2016 [63]
clinical trial	Matcha tea (2 cups a day)	End of the 30 days, improving oral health-related quality of life in patients with gingivitis	Abdul-Wahab et al., 2024 [54]
*in vitro*	Matcha tea extract (5 and 10 μg/mL)	In ductal breast cancer cell lines (T47D), PPAR-γ expression, membrane permeability ↑, and disrupted cancer cell metabolism	Keckstein et al., 2018 [64]
*in vitro*	Matcha tea aqueous extract (100 mg/mL)	ATP Luminescence Test: MDA-MB-231 and MCF-7 < 2% MTT Test: MCF-7%32.4, MDA-MB-231, %54.4 Neutral Red Test: MCF-7, %13.3 MDA-MB-231, %25.8	Schröder et al., 2018 [65]
	Matcha tea 70% ethanolic extract (100 mg/mL)	ATP Luminescence Test: MDA-MB-231 and MCF-7 < 2% MTT Test: MCF-7, %47.1 MDA-MB-231, %55.1 Neutral Red Test: MCF-7, %45 MDA-MB-231, %55.1	
*in vitro*	Matcha tea aqueous extract (0.2 mg/mL)	Breast cancer stem cells: Inhibition of cancer stem cell propagation	Bonuccelli et al., 2018 [66]
*in vitro*	Matcha tea aqueous extract	In WERI-Rb-1 cell lines, Apoptosis ↑. The cell cycle is arrested, IC_50_: 13.3 ± 1.4 µg/mL	Sayuti et al., 2023 [33]

**Note**: ↑ indicates an increase in the measured parameter; ↓ indicates a decrease.

**Table 3:** The effects of matcha tea on the gut microbiota, diabetes, and metabolic parameters.

Study Design	Types of Matcha Tea Forms	Main Findings	Numbers of Participants	References
*in vivo*	Matcha tea ethanolic extract	Suppression of neuroinflammation, Inflammatory cytokines (TNF-α, IL-6, and IL-1β) ↓ Inducing the JAK2/STAT3 pathway in the hypothalamic arcuate nucleus	40 male C57BL/6 mice (divided into 5 experimental groups: normal control, high-fat-diet control, and three matcha ethanolic extract dosage groups, *n* = 8 per group).	Zhou et al., 2020 [69]
*in vivo*	Catechin-rich, low-quality matcha tea	Inhibition of carbohydrate absorption	32 male Wistar rats (divided into 4 treatment groups to measure carbohydrate absorption and blood glucose responses, *n* = 8 per group).	Nakamura et al., 2018 [37]
*in vivo*	Matcha tea (100–200 mg/kg, 16 weeks)	Protective effect on kidney and liver damage caused by diabetes	18 male rats (12 diabetic OLETF, 6 non-diabetic LETO; *n* = 6/group)	Yamabe et al., 2009 [70]
*in vivo*	Matcha tea aqueous extract	Adipocyte length ↓; glucose levels ↓, TG, TC, ALT, AST levels, and LDL/HDL ratio ↓ NAFLD-related proteins ↓ CYP450 activity ↑	25 male C57BL/6 mice (5 groups, *n* = 5/group)	Zhou et al., 2021 [73])
*in vivo*	Matcha tea catechin-rich samples	Fat accumulation ↓ healing effects on the lipid and glucose levels	20 male C57BL/6J mice (4 groups, *n* = 5/group)	Wang et al., 2022 [74]
clinical	Matcha tea form (once a day, 12 weeks)	Fasting blood sugar ↓ Body mass index ↓ HDL-C ↑, Lipid levels ↔ IL-10, bazophil, Hb1Ac levels ↑	40 enrolled (34 completed: 17 matcha, 17 control)	El-Elimat et al., 2022 [75]
clinical	Matcha tea form (4x1 day, 1 day)	Fat oxidation ↑	13 females (randomized, crossover design)	Willems et al., 2018 [76]
clinical	Matcha tea powder (3 g/day, 21 days)	Fat oxidation ↑ Carbohydrate oxidation ↓	12 females (randomized, double-blind, crossover design)	Willems et al., 2021 [77]
clinical	Matcha tea powder (1.5 g capsule, 14 days)	Healing gout microbiota flora beneficial bacteria *Coprococcus* ↑ pathogenic bacteria *Fusobacterium* ↓	33 healthy subjects (10 males, 23 females; 17 matcha, 16 placebo)	Morishima et al., 2023 [78]
clinical	Matcha tea (2 g/day, 14 days)	Healing gout microbiota flora Cardioprotective effects	20 healthy adults (randomized crossover design)	Takegami et al., 2022 [79]
clinical	Matcha tea (1.5 g/day, 2 months)	Leg strength, muscle weight, ↑ improved recovery, fatigue ↓, positive effect on microbiota	36 older adults (18 matcha, 18 placebo)	Shigeta et al., 2023 [80]

## Data Availability

No new data were created or analyzed in this study. Data sharing is not applicable to this article.

## Abbreviations

The following abbreviations are used in this manuscript:

AhR	Aryl hydrocarbon receptor
ALT	Alanine aminotransferase
AMPK	AMP-activated protein kinase
AST	Aspartate aminotransferase
ATP	Adenosine triphosphate
CNS	Central nervous system
COX-2	Cyclooxygenase-2
EGC	Epigallocatechin
ECG	Epicatechin gallate
EC	Epicatechin
EGCG	Epigallocatechin-3-gallate
GABA	*γ*-Aminobutyric acid
GC	Gallocatechin
GCG	Gallocatechin gallate
GSH	Glutathione
GPx	Glutathione peroxidase
HbA1c	Glycated hemoglobin
HDL	High-density lipoprotein
HFD	High-fat diet
HPA	Hypothalamic-pituitary-adrenal
HPLC	High-performance liquid chromatography
IL	Interleukin
LDL	Low-density lipoprotein
MDA	Malondialdehyde
NAFLD	Nonalcoholic fatty liver disease
NF-κB	Nuclear factor kappa B
PCA	Principal component analysis
PLS-DA	Partial least squares discriminant analysis
PM2.5	Particulate matter with an aerodynamic diameter ≤ 2.5
PPARγ	Peroxisome proliferator-activated receptor gamma
ROS	Reactive oxygen species
SCFA	Short-chain fatty acid
SIRT1	Sirtuin 1
SOD	Superoxide dismutase
STAT3	Signal transducer and activator of transcription 3
TNF-α	Tumor necrosis factor alpha
